# Girls Get WISE—A programming model for engaging girls^+^ in STEM

**DOI:** 10.3389/fpsyg.2022.924943

**Published:** 2022-12-19

**Authors:** Tamara A. Franz-Odendaal, Sally Marchand

**Affiliations:** Department of Biology, Mount Saint Vincent University, Halifax, NS, Canada

**Keywords:** outreach, middle school, science—general, STEM program, girls, science

## Abstract

The majority of STEM disciplines in Canada are male-dominated and there is a significant lack of programming available to girls. The Girls Get WISE program is a university-based program that is funded by the federal government, the university, and corporate sponsorship. This program is delivered in person by educational professionals, science students, and past participants. By engaging girls in hands-on interactive STEM activities in a safe and fun space, this program provides an opportunity for young women to showcase their talents and excitement for science-based topics. The features of this program and its evaluation over a 10 year period are described here.

## Introduction

The Girls Get WISE programs began in 2011 at a university in Halifax, Nova Scotia, Canada. The city of Halifax has a population of about 450,000 people and is located on the East coast of Canada. The university that delivered the Girls Get WISE program has a long-standing reputation for the advancement of women. At the time when this outreach program was started, there was very limited STEM programming in the region, and only one other non-profit organization running STEM programming exclusively for girls, and that program was primarily focused on getting girls interested in Trades and Technology careers. The limited number of other STEM programs in the region are mixed gender and it was clear that, in most, the percentage of girls participating was 10% or less. The university itself had no science outreach programs.

### Context

Statistically in Canada, the percentage of women working in STEM careers in Canada is low and is in stark contrast to the almost equal gender split in the labor workforce (Pereault et al., 2018). This percentage is not surprising given that male Science, Technology, Engineering, and Mathematics (STEM) graduates in Canada are more likely than female STEM graduates to work in STEM (Frank, 2019). In the 2016 Statistics Canada census, a third of men (37.5%) with a Bachelor’s degree had studied in STEM whereas only 15.3% of women with a Bachelor’ degree studied in STEM. Within STEM disciplines there are large differences in participation. For example, while over half of the men (52.2%) with a STEM Bachelor’s degree studied engineering or engineering technology, only 25.4% of women selected these fields. The percentage of professional women engineers across the country has remained steady at around 17%–20% for several years (Engineers Canada, 2020). Over the last 10 years, in all STEM disciplines, only small shifts in the percentages of women’s participation have occurred. Among university students in Canada, the percentages of women across disciplines similarly vary dramatically. Again, within Biology, female students account for about 62% of students, while only 30% in Physics (Pereault et al., 2018). University and government institutions are only starting to collect and report intersectional data to explore the participation of racialized women, indigenous women, and disabled women in these fields.

The career progression to achieving a STEM career starts with elementary and secondary school. In Canada, most public schools are mixed gender with the majority of students attending public schools. The Science curriculum across the country is varied with each province regulating and determining course content. Science is taught as a single subject from elementary through to junior high school in Nova Scotia, Canada. In high school (grades 10–12), students can select Biology, Chemistry, and/or Physics. Mathematics is required in each year of schooling from primary through to grade 12, however the amount of math (one to three courses) varies depending on the student’s interests. Science teachers have limited time and resources to conduct hands-on activities in the classroom, especially in junior high (Grades 7–9) and high school (Grades 10–12). Grade 9 of high school is when students begin to select courses for the following school year and this is when students are either selecting or not selecting science-based courses. The choices made in Grade 9 for Grade 10, dictate the science options available to them in Grade 11 and Grade 12. All Science degree programs in Canada require Science pre-requisite courses from high school. Therefore a student without science subjects in Grade 12, has to spend an extra year or two obtaining these course credits before they are accepted into university science programs. Furthermore, university education is very expensive in Canada and is not affordable to many students. Thus, girls who pursue STEM-based programs at university are often those who (i) are encouraged by science and mathematics teachers and/or parents; (ii) are from affluent families who can afford university tuition and tutors (if needed) during high school; and (iii) who are exposed to STEM careers through family members. Programming, such as the Girl Get WISE events, is needed because the STEM stereotype is heavily white male-dominated in Canada and is reinforced by the branding of toys, clothing for children, and the voices/images portrayed in the media (e.g., Steinke, 2017). Thus, while many girls are interested in STEM subjects, they cannot see themselves in these careers because of the strong stereotype that still exists. Girls also have limited opportunities to learn that there are other girls interested in these subjects and that you do not have to be the top student in their science and mathematics class to have a career in STEM. Previous research has shown that girls tend to be most interested in careers that help society (e.g., Heaverlo et al., 2013; Franz-Odendaal et al., 2020; Aviolo et al., 2022). STEM professionals, the media, and STEM organizations need to do a better job at portraying their fields as helping society and to shift the additional stereotype of this as lonely work.

## Materials and methods

### Programming overview

The two signature Girls Get WISE events described below are a one-day Girls Get WISE Science Retreat and Junior and Senior Girls Get WISE Science Summer Camps. These events are open to individuals who identify as girls (i.e., girls+). Both of these events feature hands-on STEM activities as well as an hour-long Role Model session. The hands-on sessions are always very interactive and ensure the girls are learning some aspect of STEM in a novel and fun manner. The hands-on activities are either developed in-house by a team of science-trained professionals or by others (i.e., science organizations or graduate students, etc.). In the latter case, all activities are thoroughly reviewed prior to accepting them into the program content (see Discussion). The Role Model session works round-robin style; five to six women working in different male-dominated STEM fields are invited to chat informally with small groups of participants about their careers for about 8 min, and then the girls switch to the next role model. Included with the recruitment of potential role models is a “Role Model Guide” guide document, which details how the session will run, as well as topics they are encouraged to cover such as their career pathways, what motivated them to pursue their careers, what their typical day is like, any setbacks they had along the way, etc. The girls are encouraged to ask questions, and question prompt cards are provided to them at the tables. Participants are informed that they can always reach out to the Program Coordinator to connect with specific role models if they have further questions after the session. It is important for girls to see people like themselves working in male-dominated fields to show them that these careers are possible.

The one-day Girls Get WISE Science Retreat brings together 50–60 girls+ in grades 7–10 (ages ~12–16 years) together at Mount Saint Vincent University (Nova Scotia, Canada) to participate in two hands-on STEM sessions and a Role Model session. The day starts with a team ice-breaker activity, which is typically an engineering design challenge or STEM trivia, and then the girls are split into two random groups to participate in their first hands-on activity of the day which is typically an hour in length. There is a break for an hour lunch (that is provided), then they move to the second hands-on activity of the day, which is then followed by the hour-long Role Model session. At least one of the hands-on activities takes place in a laboratory setting each year, and the other session could be coding or Engineering related. The day ends with some reflection, an evaluation, and prize draws.

**Figure 1:**
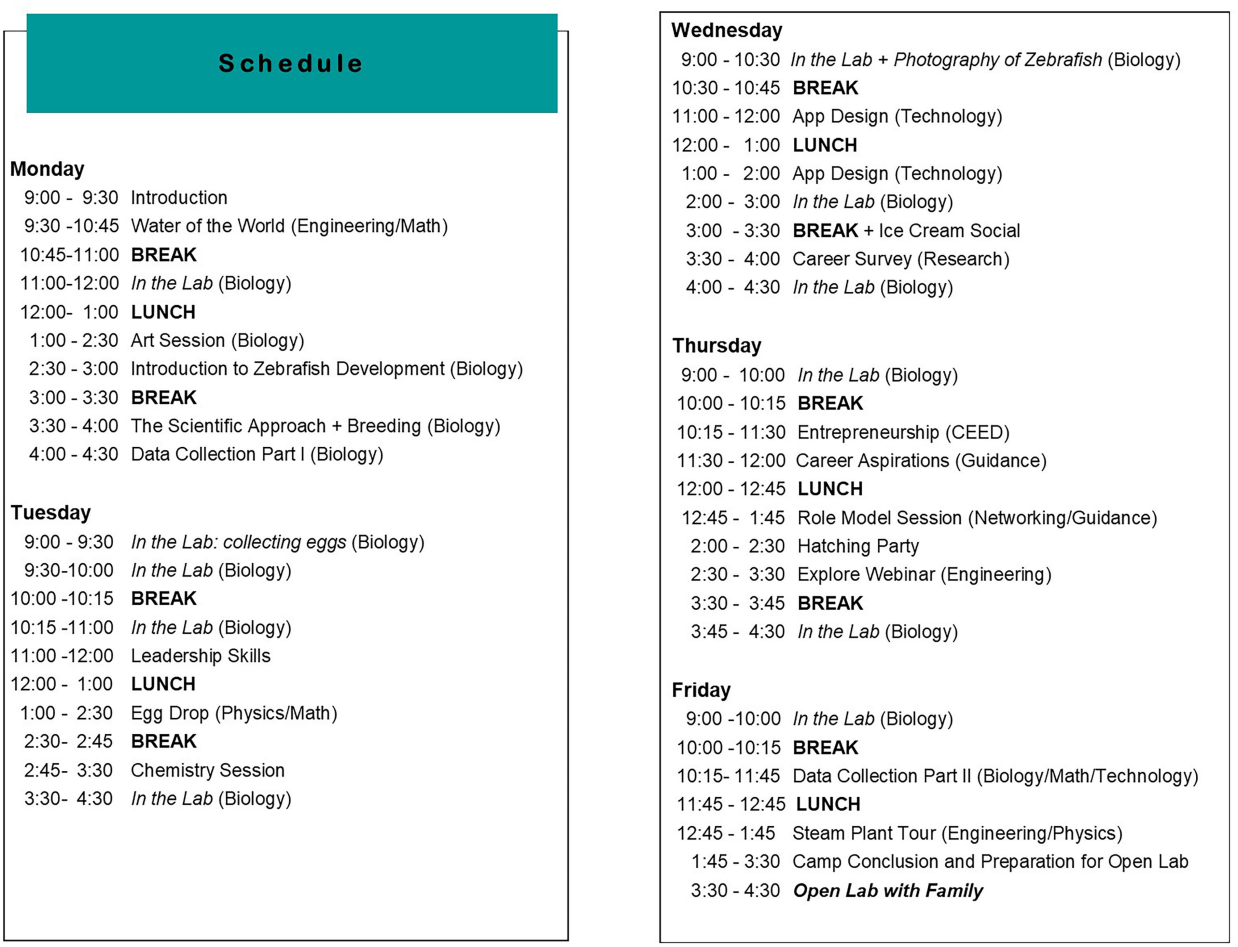
Example of a Junior Girls Get WISE Science Summer Camp program of events.

From 2012 to 2016, one Girls Get WISE Science Summer Camp took place each year for 24 girls+ that were 12–14 years in age. In 2017, a second Science summer camp for older girls, 15 and 16 years in age, was added. Both camps are day camps; however, the Junior camp runs for a full 5 days, while the Senior camp runs 4 days a week and at reduced hours per day. The difference in length is because the younger girls are more likely to depend on parents or caregivers for transport to the camp, while older girls are more able to take public transport. Similar to the Science Retreats (described above), both of the camps feature an hour-long Role Model Session, as well as different hands-on STEM activities, some of which take place in the laboratory, while others take place outdoors. Figure 1 shows an example of one of the programs for a science camp.

The major difference between the Junior and Senior camps is that the Junior camps introduce the participants to the field of Developmental Biology or Embryology through the study of zebrafish embryos. The reason for this is that the program chair is a researcher studying zebrafish development and as such these fish areas are available on campus. Zebrafish (*Danio rerio*) are wonderful organisms to showcase to students as they are easily obtainable (e.g., from pet stores), they breed readily and produce hundreds of eggs per clutch, their development is external to the mother, and their embryos are transparent (Wilk et al., 2018). The camp participants are first taught the traits of the zebrafish that make them an ideal model organism, the environmental parameters needed for their survival, and how to handle the embryos. Participants are then given several embryos from a clutch of embryos to study for the duration of the week. This teaches the participants the importance of observation and note-taking in science. The girls are provided lab-books to take notes and to draw the embryos on a daily basis. It also allows the girls to become comfortable in a laboratory setting within a university. Participants in the Junior camp spend about 2 h a day in the lab observing and caring for their zebrafish embryos. After this, the hatched larvae are returned to the researcher’s fish facility.

Another aspect of the Junior Girls Get WISE Science Camp is on the final day of camp, participants are encouraged to invite their families, friends, and community members to campus for an “open house.” Participants display STEM activities that they completed throughout the week and then showcase these to the guests. Parents are one of the key influencers on youth’s decision to pursue a STEM career or not (e.g., Dasgupta and Stout, 2014; Franz-Odendaal et al., 2016), so it is critical that parental figures are incorporated into the program. This is done by showcasing the work and projects the students have completed in the camp to parents and grandparents (and other family members and guardians) on the last day of camp. These individuals thoroughly enjoy seeing the projects that they have heard about all week from their daughters.

Other than the zebrafish component, the Junior and Senior camps have similar activities, although each is geared to the appropriate age group. On the first day of both camps there is an icebreaker activity, then a group engineering design challenge. This is done because these activities promote teamwork and critical thinking, and provide an informal opportunity to meet the other girls+ in the camp. We strive to create a program that has a balance of different Chemistry, Biology, Physics, and Technology activities throughout the week. This includes a balance between indoor and outdoor activities. The university campus is fortunate to have a beehive and beekeeper, as well as a community garden, so activities involving those resources are often developed. Students are kept very busy throughout the camp and the level of excitement increases on a daily basis.

### Pre-planning and delivery

Program development for the Science Retreats and Camps is led by the Program Coordinator with assistance from part-time staff, which in most cases are past participants of the programs. The Program Coordinator has degrees in both Science and Secondary Education. Past participants can apply to volunteer at a camp the following year. If they are interested in assisting thereafter then they are paid a standard student rate. Programming development typically begins 4 to 5 months in advance for Retreats and Camps. After selecting a date and booking the required spaces on campus. The promotional materials, which are used on social media, are prepared. These are also sent directly to Junior and Senior high schools in the area, as well as past participants. Registration is online and is first-come, first-served. It is often the case that a waiting list is required due to the high demand.

The cost for these events are very low fees: C$10 per participant for the Retreat and C$100 for the Camps. Advertisements also state that if cost is a barrier, then guardians can contact the Program Coordinator directly for a fee waiver. The aim is to make these experiences available to those of all socioeconomic backgrounds as research in both the United States (Afterschool Alliance, 2021) and Canada (Duodu et al., 2017) indicates that the cost of programming is one of the barriers youth from low-socioeconomic backgrounds face when it comes to attending after-school STEM programming. The registration fee does not cover all the costs to run the program and was implemented as a commitment to attend rather than as cost recovery. The registration fees are used to purchase materials for the activities, prizes for the participants, and thank you gifts for role models and volunteers.

The program continually recruits role models for the Role Model Sessions through a sign-up form on the program’s website, word-of-mouth, social media posts, local universities, partnerships with industry, and not-for-profit organizations. If someone is interested in participating as a role model for the program we add them to the database of role models. When it comes time to plan a Girls Get WISE event, role models are selected from this database. We prioritize role models that have not participated recently (this reduces role model fatigue), that work in different STEM fields (to ensure a diversity of careers and career paths are showcased), and that come from diverse backgrounds (e.g., racialized women). Invitations to participate are sent to five or six role models. Sometimes, the timing of the event does not align with their schedule, for example, and in these cases, our role model database is consulted once again. We found that it was extremely useful to have a database of role models we could call upon at any time since a number of repeat participants attend the events, and therefore, both the program content and the selection of role models need to be different in consecutive years.

Delivery of the majority of the hands-on sessions is carried out by the Program Coordinator, part-time students and/or volunteers. Often experts are invited to discuss their research with participants and to then lead related hands-on activities, this typically occurs if the team is not familiar with a particular STEM topic. We also invite other STEM-related non-profit groups to host activities, such as the local Science Centre or Canada Learning Code. As noted earlier, it is important that the Program team reviews the activities to ensure that they are age appropriate.

## Results

### Program impacts

At all of these Retreats and Camps, evaluations are handed out at the end of the programming on the final day. These evaluations are primarily used to determine which sessions were well received and which need improvement. We also ask some program impact questions, although we acknowledge that these are leading questions. The past participants (from the last 10 years of programming) that completed program evaluations described below range in age between 12 and 16 years of age, they all identify as young women (girls+), and they come from varied socioeconomic backgrounds. The majority of respondents live in urban areas (roughly 85%). In total, 678 girls have participated in this program over the last 10 years and completed evaluation forms at the end of the events. Since these forms are handed out in the last few hours of the event, the response rate is high (95%). The data presented below is aggregated data from the last 10 years.

One of the questions participants are asked is “Did this science camp (or retreat) meet your expectations?” Participants could select from the following options: Exceeded, Yes, or No. From responses, 96% of participants indicated that the event met or exceeded their expectations (ANOVA: *F* = 22.11; *p* < 0.005). To better understand if the Girls Get WISE programming has a lasting impact on participants, participants are asked: “Did attending this event inspire you to continue with science at school?” Participants could select from the following options: Yes, No, Maybe, Unsure. 93% of participants indicated that the event did inspire them to continue with science at school (ANOVA: *F* = 43.97; p < 0.005). Another question is: “How did attending this event affect your interest in science and engineering?” of which participants could choose the following options for their response: More Interest, Same Interest, Less Interest. Results from this question were as follows: 60.7% of participants indicated that the event increased their interest, while 36.9% indicated that their interest stayed the same (ANOVA: *F* = 5.12, *p* < 0.05). Based on these responses, the many emails we receive after events and participant comments, it is evident that this programming style is highly successful; the longer girls stay on this science path the more exposure they would have to science and engineering as a career.

Also part of these post-event evaluations, participants are asked to rate each session overall on a scale of 1 to 5 with 1-disliked to 5-excellent. The role model session is consistently rated as one of the top sessions at these events by participants, getting an average rating of 4.3 out of 5. When asked the open-ended question “Do you have any additional comments or suggestions?” many participants mention the role model session in particular. Some comments received include: “*I would love more time to talk with the role models. They were so Inspiring*.,” “*Role models were very fun and loved to hear them*,” and “*The role model session helped to round out some questions I had about university*.”

It is clear from the event evaluations that the STEM activities that were rated the highest are also those that involve using equipment few would have access to in schools, and those that are very hands-on. A few activities that were rated 4.5 or higher out of 5 were: studying the zebrafish in the lab, making soap with a local soap maker, working with planaria, making bath bombs, microscope scavenger hunt, and budgeting for life. Three of these activities, working with zebrafish, planaria, and the scavenger hunt, all involve the use of microscopes. Planaria are a type of flatworm that are able to regenerate its tissues, in this activity participants are asked to predict what will happen when certain parts of the work are cut away. They then perform the cuts and observe what happens over several days while caring for the planaria. The microscopes scavenger hunt has participants work in pairs and use written clues that match with prepared microscopes slides to make an educated guess as to what is on the slide. At the university, working with invertebrates such as planaria or with zebrafish embryos does not require animal ethics approval.

The one activity that differs from the others in the list above is “Budgeting for life.” This activity was developed based on the game of life where participants are randomly given a job with an average salary and are asked to create a monthly budget using a template in Excel. This activity was designed so that participants were able to see the financial benefits that the majority of STEM careers can have over non-STEM career fields. Empowering the girls to be independent and financially secure women is an added benefit of a STEM career. A discussion about this activity which includes stressing that their passions and interests should be the primary determinant of their future career is conducted after the activity.

The Girls Get WISE events retain a good number of participants. On average, approximately 28% of participants attend more than one of the Girls Get WISE events. It is important to note that with a narrow age range for the events, many girls age out of the program within a year or two.

## Discussion

Since the introduction of the Girls Get WISE programs in 2011, there has been an increase in STEM programming focused on engaging girls in Halifax, across the province, and in neighboring provinces. It is exciting to see this expansion in programs, especially to more rural parts of the region where it is difficult for the programs to reach on a consistent basis. A takeaway from this increase in all-girl programming is that parents are looking for these opportunities for their daughters and that funders are seeing the benefits and want to keep the momentum going.

Throughout the 10 years of programming, we have strived to utilize the feedback gathered from participant evaluations to improve on these program offerings. For instance, if a particular hands-on STEM session is rated less than three out of five on a five-point Likert scale, then the activity is reviewed and discussed to determine ways to improve that activity with the available knowledge and resources. If it could not substantially be improved, then the activity would no longer be offered. An example is that, after receiving suggestions from several camp participants that the number of hours spent in the laboratory studying zebrafish development was a little too long, we decreased the time for these sessions for all future camps. To ensure the success of this program, it is essential that activities are evaluated on a regular basis.

In the first few years of this program, the majority of the hands-on sessions were developed by the program team, but over time, external subject matter experts from the area were recruited to develop and run some of the activities. This was done because (i) it is time consuming to develop new activities, and (ii) the activities the team wanted to run were not in their field of expertise. This approach is two-fold, participants are able to meet additional STEM experts and the activities are often more tailored on a particular topic. This approach comes with other challenges, however. First, just because someone is an expert in a particular field or topic, does not mean that they have the experience in delivering an activity to youth based on that knowledge. To address this challenge, an activity proposal sheet was developed in which prospective facilitators were asked to complete. This form asks them to detail their activity and provide all relevant materials that would go along with it. The program team then works with the facilitators to tweak the content if needed. This proved to be an effective way to develop quality and age-appropriate content for the program.

**Figure 2:**
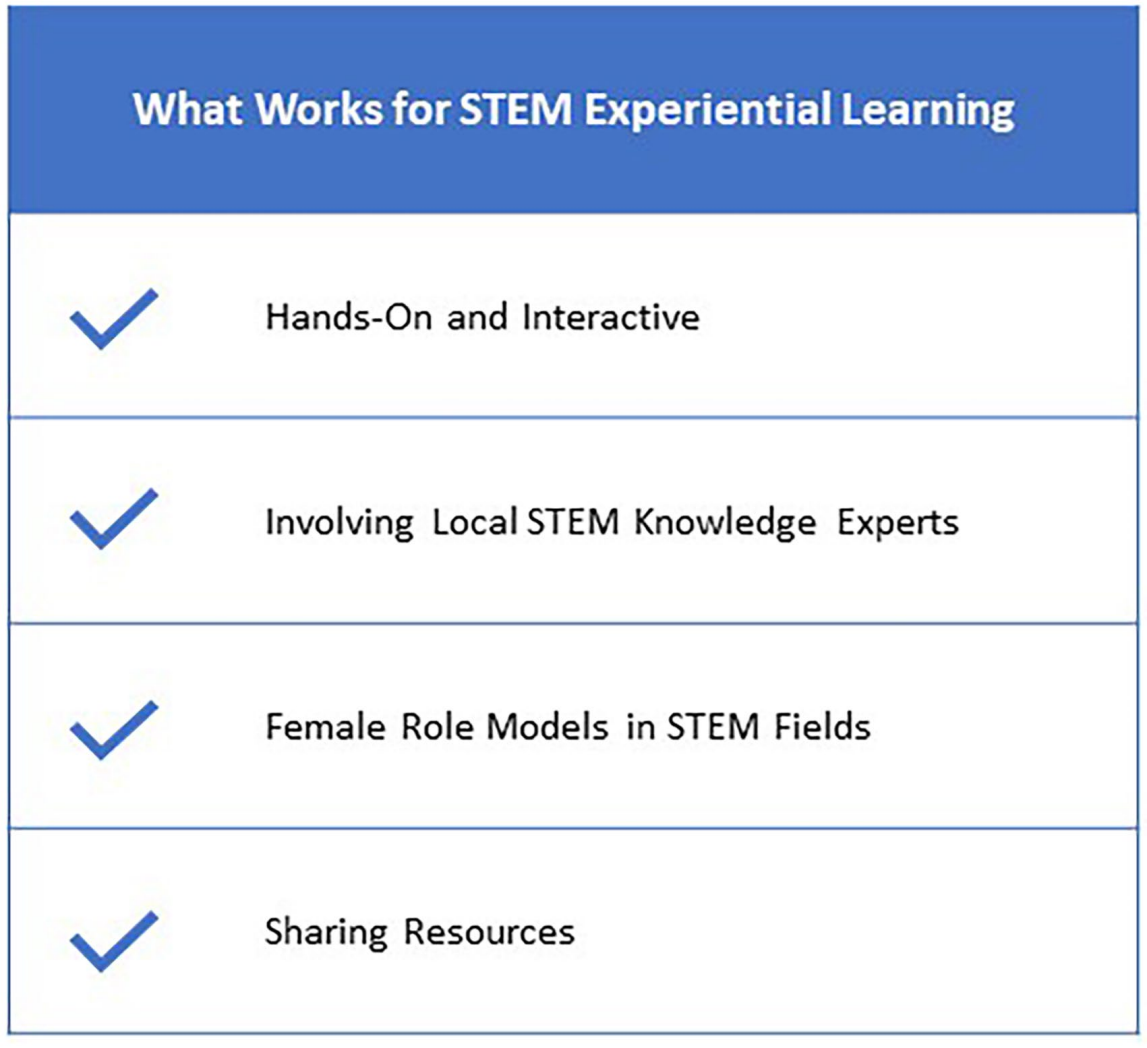
A list of “What Works” for planning and delivering STEM experiential learning events.

Some lessons that were learned along the way in delivering all-girls STEM programming over the last decade are that it is important to create activities that are as hands-on and as interactive as possible, use the knowledge of local STEM experts to develop unique programming, and include them in the delivery if possible, including female role models as part of the programming is very impactful, and always strive to improve upon programming using feedback from participants (Figure 2). Specific to the Role Model Session, the ideal time for each “round” with a role model is seven to 8 min, over that amount is too long and the participants start to fidget, and under that time does not provide enough time for meaningful connection. The retreat format is quite popular and our team often serves as a resource to other groups wishing to run similar all-day events. Sharing resources is key to expanding reach.

While the school system curricula have remained largely unchanged over the last 10 years, there is a need to depend on other organizations to encourage the pursuit of STEM. Past research has shown that girls are influenced by their parents and guardians, teachers, and their peers (e.g., Franz-Odendaal et al., 2020). When speaking to the participants, peer influence appears to be the main driver in their decision to participate or not, in a STEM camp.

To build on these programs in the future, we would recommend the creation of an ambassador’s program, where past participants become program ambassadors and through video and other social media content encourage their peers to participate in the program. Another program that would expand on the Girls Get WISE model would be to create a Girls Get WISE Leadership program, where past participants can get leadership training and then begin to volunteer with the programs. This helps to build confidence in the girls and would help to ensure a steady stream of ready and eager volunteers for STEM outreach programming.

The longer-term impact of these programs on the STEM workforce or student enrollment into STEM programs is unknown at this time. However, some insight can be gained from some of these unsolicited emails that were received: In the words of one of our participants “*I just wanted to touch base to let you know that the camps had an impact on my academic direction. I have just begun my first year in a Bachelor of Science degree in Biology at .. I wanted to thank-you for the opportunities we had during the WISE camps to better understand the different aspects of science and its various influences. It opened my eyes to different career possibilities. I am enjoying my first month at … and wanted to reach out to let you know these wonderful camps do make a difference*.”

And another email from a father of a past participant: “*My two daughters did the WISE Atlantic program a few years ago. My oldest has just been accepted to … University for Biology/Medical Sciences with a scholarship. I give great credit to your program for inspiring her to study and enjoy science. Many thanks for your program and the people who work to make it possible*.”

## Data availability statement

The original contributions presented in the study are included in the article/supplementary material, further inquiries can be directed to the corresponding author.

## Author contributions

TF-O and SM designed and delivered the programming and wrote the manuscript. All authors contributed to the article and approved the submitted version.

## Funding

Funding was received from the NSERC Chair for Women in Science and Engineering (Atlantic region). This program is funded by the Natural Sciences and Engineering Research Council of Canada (NSERC), Mount Saint Vincent University, Lockheed Martin Canada.

## Conflict of interest

The authors declare that the research was conducted in the absence of any commercial or financial relationships that could be construed as a potential conflict of interest.

## Publisher’s note

All claims expressed in this article are solely those of the authors and do not necessarily represent those of their affiliated organizations, or those of the publisher, the editors and the reviewers. Any product that may be evaluated in this article, or claim that may be made by its manufacturer, is not guaranteed or endorsed by the publisher.
